# Spatial and temporal associations between physical establishment density and hearing difficulty across US counties

**DOI:** 10.1371/journal.pone.0355504

**Published:** 2026-08-11

**Authors:** Tianle Duan, Qingchun Li, Noori Kim

**Affiliations:** 1 Bowen School of Construction, Purdue University, West Lafayette, Indiana, United States of America; 2 School of Engineering Technology, Purdue University, West Lafayette, Indiana, United States of America; University Medical Center Hamburg-Eppendorf: Universitatsklinikum Hamburg-Eppendorf, GERMANY

## Abstract

Hearing loss is a critical public health issue in the United States, affecting more than 15% of American adults. Existing studies have primarily focused on physiological and socioeconomic factors associated with hearing health. This study investigates the spatial and temporal associations between physical establishment density and hearing difficulty rates across US counties. Bivariate correlation analysis shows that counties with denser physical establishments generally exhibit lower hearing difficulty rates, and vice versa. Counties with lower hearing difficulty rates also tend to exhibit denser road networks, higher socioeconomic status, younger population structures, better insurance coverage, and higher educational attainment levels. After controlling for road density and other sociodemographic covariates in regression analyses, physical establishment density becomes positively associated with hearing difficulty rate. Our analyses indicate that road density largely drives this sign reversal and reflects broader infrastructure accessibility and urban service connectivity. We also identified hearing-health-risky physical establishments, representing environments potentially associated with elevated occupational or lifestyle-related noise exposure. Hearing-health-risky physical establishment density showed a stronger positive association with hearing difficulty rates than overall physical establishment density. Temporal clustering analysis further reveals that counties exhibiting increasing trends in hearing difficulty rates during 2012–2021 tended to have higher physical establishment density. Our findings suggest that counties characterized by denser built environments and better socioeconomic conditions currently exhibit lower hearing difficulty rates at the aggregate level, while urbanized environments may also be associated with growing long-term hearing-health risks. These findings provide implications for hearing-related urban planning, environmental noise management, and public health policy.

## 1 Introduction

Hearing difficulty (HD) is one of the fourth leading cause of disability in the world and remains one of the most prevalent chronic medical conditions in the United States [1,2]. HD affects more than 15% of Americans aged 18 and older, and the affected population is projected to double by 2060 [2,3]. It leads to social isolation, depression, cognitive decline, and other negative health outcomes (e.g., brain disorders), which adversely affect the quality of life [3,4]. HD can exacerbate social inequality by shaping individual economic status, with studies showing that the HD population has a lower income than the comparison group [5]. HD also causes huge economic implications for the US due to reduced productivity, lower employment and wages, and increased costs in healthcare and special education [6,7].

Existing studies on HD have widely found its connection to physiological and socioeconomic factors. From a physiological perspective, age-related Hearing Loss (AHL) is a major form of HD, resulting from cochlear degeneration with the cumulative effects of extrinsic damage (e.g., noise and other ototoxic agents) and intrinsic disorders [8,9]. Individual genetic factors (e.g., mutations in certain genes) determine the timing and severity of AHL [8,10]. Gender is also an influencing factor, evidence showing that men’s hearing sensitivity declines faster than women’s at most ages, resulting in a predominance of HD in men [11–13]. In addition, HD is associated with risk factors caused by unhealthy lifestyles, such as diabetes, hypertension, and smoking [14]. From a socioeconomic perspective, existing studies show that socioeconomic factors such as income level, insurance coverage, and educational attainment have a statistically significant correlation with hearing health [15–17]. These factors serve as important confounders when researching other HD-related factors.

Physical establishments (PEs), which refer to the built environment features such as commercial buildings, retail outlets, and service facilities, often serve as a multifaceted indicator in urban studies [18]. PE density can indicate service accessibility via spatial proximity to amenities. A well-developed establishment system improves human well-being by providing essential services such as clean water, sanitation, transportation, healthcare, and entertainment, all of which are fundamental to improving quality of life and supporting socioeconomic development [19].

From another perspective, PE density represents exposure levels in noise by capturing population activity intensity across space [18,20–22]. Occupational noise exposure in workplace environments is an important contributor to HD [23]. Noise-induced hearing loss (NIHL) is one of the most common forms of acquired hearing impairment in industrialized countries and often develops following prolonged exposure to high sound levels to damage auditory function [24]. Previous studies have reported that the highest prevalence of self-reported occupational noise exposure occurs in the Mining, Construction, Manufacturing, Utilities, and Transportation and Warehousing industries [25]. Workers employed in these sectors also exhibit significantly higher risks of HD compared with workers in the Finance and Insurance sector [25]. Lifestyle-related establishment noise exposure is also an increasing public health concern [26]. Growing evidence suggests that individuals are increasingly exposed to unsafe sound levels in recreational and lifestyle-related environments, including nightclubs, bars, cinemas, concerts, live sporting events, and fitness classes [27]. Previous studies have estimated that approximately 40% of the adolescents and young adults (12–35 years old) in middle- and high-income countries are exposed to damaging sound in entertainment venues such as clubs, discotheques, and bars [26,27]. Therefore, identifying the mechanism behind HD disparities and understanding the overall impact of PEs are crucial for policy-making and urban planning to improve hearing health and quality of life.

On one hand, dense urban environments can produce high levels of noise that directly cause HD and decrease people’s overall satisfaction with their quality of life [28]. On the other hand, studies on urban-rural health disparities argue that limited access to health facilities or services in rural areas negatively affects hearing health and overall public health outcomes [29]. Existing studies stemmed from different impacts of PEs, revealing conflicting effects of ‘ease or noise’ on public health [30,31]. Therefore, there remains a critical gap in understanding the overall associations between PEs and HD at large spatial scales.

To fill the research gaps, this study uses a big-data-driven approach to investigate the spatial and temporal associations between PEs and HD. In this study, we define PEs as points of interest (POIs) provided by the SafeGraph Global Places & Geometry, which records geocoded business using North American Industry Classification System (NAICS) codes [32]. We first used PE density as a proxy for urbanization intensity and human activity intensity to investigate its association with HD rates across U.S. counties. In addition, hearing-health-risky PEs are places where individuals are more likely to experience noise exposure through occupational activities or lifestyle-related environments. We therefore further identified hearing-health-risky PEs and incorporated them into the analysis to capture a potential environmental noise exposure pathway.

## 2 Data and methods

### 2.1 Data definition and pre-processing

#### 2.1.1 Scale of analysis.

We conducted the analysis using county-level data. The distances of people’s daily travel ranges provide information on their interactions with their environment [33]. Evidence shows that the median range of daily mobility for US people is approximately the size of a typical county [34]. This study used publicly available county-level Census data and licensed commercial point-of-interest data, with no individual level identifiers. Therefore, the data use does not require informed consent.

#### 2.1.2 Hearing difficulty rate.

We sourced HD data from American Community Survey (ACS) 5-year estimates (Table B18120-5Y) from the US Census Bureau. This dataset records the population with deafness or serious difficulty hearing. We used the HD rate to evaluate a county’s HD level, accounting for differences in population size across counties. We calculated the HD rate by dividing the population with HD by the total population of a county. In this study, we used the HD rate from 2012 to 2021 for a time series analysis. We linked the HD rate in 2021 to PE density for a cross-sectional regression analysis.

#### 2.1.3 Physical establishment density.

We sourced PEs data from the SafeGraph Global Places & Geometry dataset through the Dewey Data platform [32]. The dataset is compiled and maintained by the SafeGraph company and updated monthly. In this study, we sourced existing PEs in 2021 from this dataset to be consistent with our cross-sectional analyses based on the 2021 ACS 5-year hearing difficulty estimates. We included all types of PEs recorded in this dataset when calculating PE density. This data records PEs’ geographic coordinates, brands, and categories labeled with the North American Industry Classification System (NAICS) codes. We extracted hearing-health-risky establishments by filtering specific NAICS codes based on hearing-health-risky criteria (i.e., over 85 decibels reported by the American Speech–Language–Hearing Association).

By matching establishment geographic coordinates with cartographic boundaries of US counties, we grouped all establishments into the county to which the data belong. We used PE density to remove the effect of county size. A county’s overall PE density, $D_{i}$, is defined as follows:


$$\begin{array}{c} \begin{array}{c} D_{i}=\frac{N_{i}}{A_{i}} \end{array} \end{array}$$ (1)

where $N_{i}$ is the total number of PEs in the $i^{th}$ county, and $A_{i}$ is the land area of the $i^{th}$ county.

#### 2.1.4 Road density.

We used the road network data provided by the OpenStreetMap (OSM) program to calculate county-level road density [35]. We retrieved roads accessible by vehicles and computed road density as the total road length of a county divided by the county’s total land area.

#### 2.1.5 Index of relative rurality.

We used the index of relative rurality to quantify the level of rurality of a US county [36]. The index of relative rurality is a metric that measures the extent of rurality of a region, mainly determined by population density, economic diversity, and accessibility [36]. It ranges between 0 (low level of rurality, i.e., urban) and 1 (most rural), providing a more flexible and comprehensive assessment than traditional binary classifications (urban and rural).

#### 2.1.6 Other socioeconomic and demographic data.

We used the socioeconomic and demographic variables reported by the US Census Bureau, including sex ratio, old dependency ratio, median income, insurance coverage, and educational attainment. The sex ratio is defined as the male population divided by the female population. The old dependency ratio is defined as the elderly (aged 65 or over) divided by the number of working-age people (aged 15–64), which is used to control the effects of aging.

### 2.2 Correlation analysis

We used Spearman’s rank correlation coefficient to quantify the monotonic associations between county-level HD rates and county characteristics. A Spearman’s rank correlation coefficient, $\rho_{s}$, can be computed as in equation (2), where R(X) and R(Y) denote the ranked values of variables, and $\sigma_{R(X)}$ and $\sigma_{R(Y)}$ denote their standard deviations.


$$\begin{array}{c} \begin{array}{c} \rho_{s}=\frac{cov(R(X),\;R(Y))}{\sigma_{R(X)}\;\sigma_{R(Y)}\;} \end{array} \end{array}$$ (2)

The Spearman correlation between two variables will be high (close to 1) when observations have a similar rank, indicating a strong positive correlation. It will approach −1 when they exhibit a strong negative correlation. We used the Python package *SciPy* to perform the Spearman correlation analysis.

### 2.3 Regression analysis

We established a series of regression models to examine the associations between PE density and HD rates across counties in the United States while controlling for potential confounding factors. We incorporated state-level fixed effects to account for unobserved heterogeneity across states, including differences in healthcare systems, socioeconomic structures, regulatory environments, and infrastructure development patterns. We specified the models as follows:


$$\begin{array}{c} \begin{array}{c} Y_{i,s}=\beta_{\text{1}}{PED}_{i,s}+\beta_{\text{2}}RD_{i,s}+X_{i,s}\beta +\alpha_{s}+\epsilon_{i,s} \end{array} \end{array}$$ (3)

where $Y_{i,s}$ represents the HD rate in county i within state s; $PED_{i,s}$ and $RD_{i,s}\;$denote the PE density and road density of county i, respectively; and ${𝐗}_{i,s}$ represents a vector of additional county-level covariates, including median income, old dependency ratio, sex ratio, and educational attainment. The coefficient vector β captures the associations between the explanatory variables and HD rate. The term $\alpha_{s}$ represents state-level fixed effects, and $\varepsilon_{i,s}$ captures unexplained variation not accounted for by the included variables.

We standardized all continuous variables to zero means and unit variances for coefficient comparability across variables. We clustered standard errors at the state level to account for potential within-state correlation among counties. To avoid multicollinearity problems, we filtered the variables using the Variance Inflation Factor (VIF). We conducted this cross-sectional regression analysis using the county-level HD rate and PE density in 2021. We used the R package *fixest* to perform the regression analyses.

### 2.4 Time-series clustering analysis

We conducted time-series clustering to explore the temporal development of HD rates in US counties. First, we used the silhouette score method to determine the optimal number of clusters. We then applied K-means clustering with the soft Dynamic Time Warping (soft-DTW) method to historical HD data from 2012 to 2021. This approach captures long-term trends in HD rates across counties. We subsequently conducted Mann–Whitney U tests using 2021 county-level features to investigate their associations with different HD-rate development patterns [37]. We used the Python package *tslearn* to perform soft-DTW K-means clustering.

## 3 Results

### 3.1 Bivariate correlations between HD rates and county characteristics

**Fig 1:**
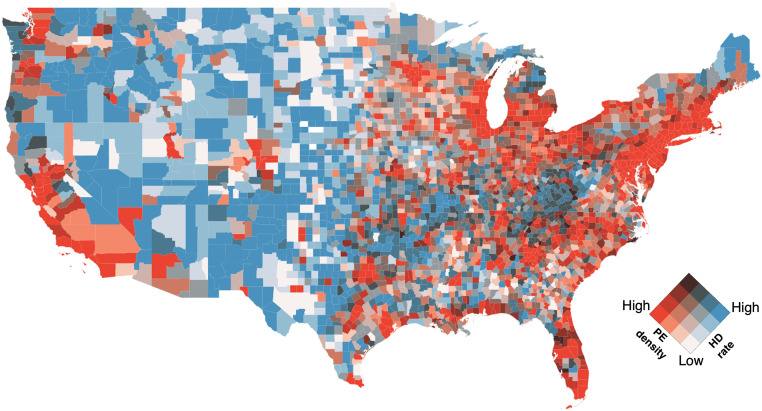
Bivariate choropleth map of county-level HD rate and PE density. Both variables are rank-normalized and classified using quartile-based categories. The color combinations represent the joint distribution of HD rate and PE density within each county. Red hues indicate counties with relatively high PE density and low HD rate, whereas blue hues indicate counties with relatively low PE density and high HD rate. Darker colors represent counties where both variables are relatively high, while lighter colors represent counties where both variables are relatively low. Counties were treated equally without population weighting. We obtained the basemap county boundaries from the U.S. Census Bureau TIGER/Line Shapefiles (https://www.census.gov/geographies/mapping-files/time-series/geo/tiger-line-file.html), which are public domain U.S. government data. We generated this figure using Python.

**Fig 2:**
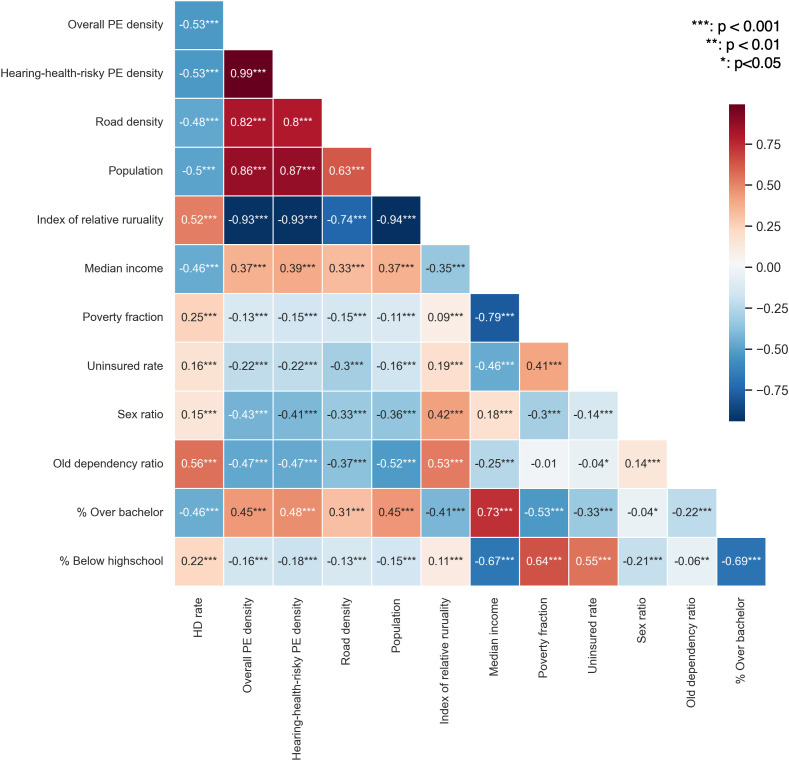
Spearman’s correlation matrix.

The bivariate choropleth map (Fig 1) presents the spatial distribution of county-level HD rate and PE density. Counties characterized by relatively high PE densities and low HD rates, as well as counties with relatively low PE densities and high HD rates, are more common across the United States. In contrast, counties exhibiting both high PE density and high HD rate, or both low PE density and low HD rate, are less common. This overall spatial pattern is consistent with the negative Spearman correlation between PE density and HD rate at the national county level (Spearman Rho −0.53, see Fig 2).

Fig 2 presents the correlation matrix, which displays the coefficients that describe the strength and direction of the correlation between each pair of variables. US counties with higher PE densities tend to show lower HD rates, and these counties also exhibit larger population and larger road density, and vice versa.

Counties with higher HD rates tend to exhibit significantly lower socioeconomic status. Specifically, county-level HD rates are strongly correlated with higher relative rurality and higher old dependency ratio (|Spearman’s ρ | ≥ 0.5), moderately correlated with lower median income, lower proportions of residents with a bachelor’s degree, and lower road density (0.3 ≤ |Spearman’s ρ | < 0.5), and weakly correlated with higher poverty rate, higher uninsured rate, higher proportion of residents with below high school education, and higher sex ratio (|Spearman’s ρ | < 0.3). Meanwhile, these factors also show statistically significant correlations with PE density. County-level PE density is strongly associated with higher road density and lower relative rurality (|Spearman’s ρ | ≥ 0.5), moderately correlated with higher median income, lower old dependency ratio, lower sex ratio, and higher proportions of residents with a bachelor’s degree (0.3 ≤ |Spearman’s ρ | < 0.5), and weakly correlated with lower poverty rate, lower uninsured rate, and lower proportions of residents with below high school education (|Spearman’s ρ | < 0.3). Because these variables are associated with both PE density and HD rates, they represent potential confounding factors and are therefore included as covariates in the subsequent regression analyses.

### 3.2 Multivariate regression analysis of HD rates

We conducted cross-sectional regression analyses following the framework described in Section 2.3. In the previous bivariate correlation analysis, counties with higher PE densities generally exhibit lower HD rates $\left(Spearman^{\prime }s\;\rho =-\text{0}.\text{5}\text{3},\;p<\text{0}.\text{0}\text{0}\text{1}\right)$. Model 1 in Table 1 presents the crude association between PE density and HD rates (β = −0.006, SE = 0.004). After controlling for sociodemographic covariates and state-level fixed effects (see Model 2 in Table 1), the association between PE density and HD rate remains negative but is not statistically different from zero (β = −0.001, SE = 0.014).

**Table 1:** Regression results.

Variable	Model1	Model2	Model3	Model4
**PE density**	−0.006 (0.004)	−0.001 (0.014)	0.114* (0.046)	
**Hearing-health-risky PE density**				0.125* (0.049)
**Road density**			−0.264*** (0.069)	−0.277*** (0.073)
**Median Income**		−0.210* (0.097)	−0.209* (0.096)	−0.212* (0.096)
**Old Dependency Ratio**		0.909*** (0.095)	0.868*** (0.095)	0.867*** (0.095)
**Sex Ratio**		0.011 (0.073)	−0.017 (0.074)	−0.018 (0.074)
**Over Bachelor**		−0.615*** (0.084)	−0.530*** (0.088)	−0.530*** (0.088)
**Below High School**		0.034 (0.127)	0.056 (0.128)	0.055 (0.128)
**R** ^**2**^	0.203	0.495	0.499	0.499
**AIC**	13371.46	11967.52	11941.56	11940.58
**N**	3103	3103	3103	3103

Main values are regression coefficients. Standard errors are reported in parentheses.

***: p < 0.001, **:p < 0.01, *:p < 0.05.

Road density largely drives a sign reversal in the association between PE density and HD rate. After additionally controlling for road density (see Model 3 in Table 1), PE density becomes positively associated with HD rate. Specifically, a one-standard-deviation increase in PE density is associated with a 0.114-standard-deviation increase in HD rate (SE = 0.046, p < 0.05). Meanwhile, road density shows a negative association with HD rates, with a one-standard-deviation increase in road density associated with a 0.264-standard-deviation decrease in HD rate (SE = 0.069, p < 0.001). In Model 4, we further incorporated hearing-health-risky PE density (see S1 Note for identification criteria). The results showed that a one-standard-deviation increase in hearing-health-risky PE density is associated with a 0.125 standard deviation increase in HD rate (SE = 0.049, p < 0.05), indicating a statistically significant positive association after controlling for road density and socioeconomic covariates. We conducted sensitivity analyses using different categories of hearing-health-risky PEs. We provided the full results in S2 Note.

Several additional covariates also showed statistically significant associations with HD rate. In Model 3, the old dependency ratio is positively associated with the HD rate (β=0.868, SE=0.095, p<0.001), whereas median income (β=−0.209, SE=0.096, p<0.05) and the proportion of residents with a bachelor’s degree are negatively associated with HD rate.

### 3.3 Temporal clustering patterns of HD rates from 2012 to 2021

**Fig 3:**
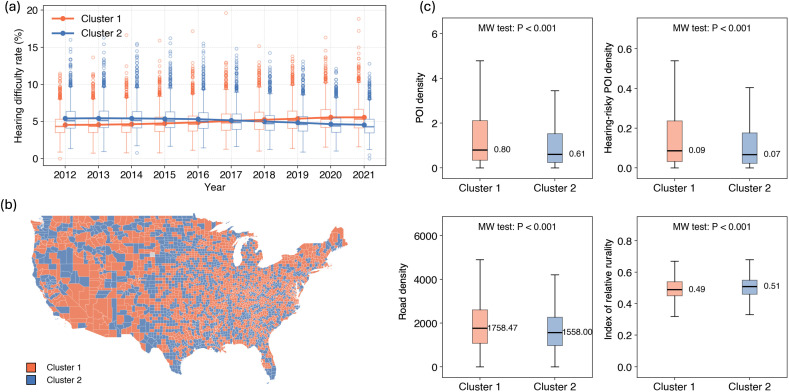
Time-series clustering: (a) Temporal clustering results derived from soft Dynamic Time Warping (soft-DTW) K-means clustering. (b) Spatial distribution of the two temporal clusters across U.S. counties. We obtained the basemap county boundaries from the U.S. Census Bureau TIGER/Line Shapefiles (https://www.census.gov/geographies/mapping-files/time-series/geo/tiger-line-file.html), which are public domain U.S. government data. We generated this figure using Python. (c) Mann–Whitney U test comparisons between the two clusters.

Fig 3 illustrates the time-series clustering results (see S3 Note for technical details of the soft-DTW implementation). The 3,102 counties (without missing historical data) exhibited two temporal clusters and patterns in HD rates during 2012–2021. A total of 1,746 counties showed an increasing HD-rate trend (Cluster 1 in Fig 3), whereas the remaining 1,356 counties exhibited a decreasing trend (Cluster 2 in Fig 3). We used the Mann–Whitney U test (MW test) to examine differences between the two clusters. As shown in Fig 3c, counties in Cluster 1, which exhibited increasing HD-rate trends, had statistically significantly higher overall PE densities (median: 0.80 vs. 0.61 establishments per square mile, approximately 31% higher), hearing-health-risky PE densities (median: 0.09 vs. 0.07 establishments per square mile, approximately 29% higher), and road densities (median: 1758 vs. 1558 m per square mile, approximately 13% higher) than counties in Cluster 2. In contrast, the difference in the index of relative rurality was comparatively modest (median: 0.49 vs. 0.51, approximately 4% lower). Although the absolute difference in hearing-health-risky PE density was modest (0.09 vs. 0.07 establishments per square mile), it represented an approximately 29% higher median density in Cluster 1 than in Cluster 2. This difference should be interpreted cautiously, but it should not be dismissed. Because this measure is expressed as a density, a small per-square-mile difference may accumulate across county areas and reflect differences in population-level exposure environments. These results suggest that counties with increasing HD-rate trends tended to exhibit denser built-environment characteristics at the population level. Considering that the ACS-1Y estimate HD data has missing values for US counties (1-year estimation only captures population larger than 65,000), our time-series clustering analysis used ACS 5-Y estimation HD rate, which therefore reflects long-term temporal patterns rather than precise year-to-year changes.

## 4 Discussion

This study uses PE density as a proxy for urbanization intensity and human activity intensity and further incorporates hearing-health-risky PEs to capture a potential environmental noise exposure pathway [38,39]. At the bivariate level, PE density was negatively correlated with HD rate, indicating that counties with higher PE densities generally exhibited lower HD rates. This pattern may reflect urban-rural structural differences, as counties with higher PE densities also tend to be more urbanized, economically developed, and supported by denser infrastructure and greater healthcare accessibility. These aggregate urban advantages are associated with lower HD rates at the county level and therefore contribute to the observed negative bivariate association.

However, after controlling for road density and other socioeconomic and demographic covariates, including income, rurality, education, and insurance-related factors, the coefficient of PE density became positive. Our analyses also indicated that road density largely drove this sign reversal, and road density itself was negatively associated with HD rates. Road density reflects infrastructure accessibility and urban service connectivity, including access to healthcare services, transportation systems, screening opportunities, and other socioeconomic resources [40]. After accounting for these accessibility-related advantages associated with urbanization, the remaining variation in PE density may reflect other dimensions of concentrated urban activity, including environmental exposure intensity, occupational structure, lifestyle-related exposure environments, and dense human activity settings, which are positively associated with HD rate in the regression analysis [15,41].

Hearing-health-risky PE density showed a stronger positive association with HD rates than overall PE density, suggesting that environments associated with elevated occupational or lifestyle-related noise exposure may be more closely associated with hearing-health disparities. In particular, categories related to manufacturing, construction, transportation and logistics, and entertainment/high-noise venues showed consistently positive associations in the sensitivity analyses. These findings support the interpretation that different dimensions of the built environment may have distinct relationships with hearing-health outcomes.

The study also showed that socioeconomic and demographic factors play a significant role in shaping HD rate disparities across US counties. Studies on HD should consider involving them as potential confounders. Existing studies recognized a feedback loop between HD disparities and economic disparities [42] that the economic position can interact in a bidirectional relationship with hearing health [43]. Counties with higher median income and lower poverty rates are more urbanized and developed, offering more advanced healthcare infrastructure and broader medical insurance coverage [44]. Low-income people tend to live in less developed areas, such as rural areas, and have less access to health resources and services (e.g., healthcare facilities and amenities). People with HDs may struggle to achieve higher educational attainment and secure high-paying jobs, making it challenging to afford living and healthcare services in developed regions [5,9]. The high cost of hearing aids makes treatment unaffordable for low-income people, reducing the HD population in urban regions [45]. Addressing socioeconomic disparities is crucial to ensure equitable access to hearing health services and resources for all people, regardless of their economic status. The lack of essential services and resources (e.g., healthcare-related facilities) in rural areas induces HD disparities between urban and rural areas [14,44]. Transportation difficulties, low education level, and financial constraints are all crucial factors affecting access to essential services and resources, increasing HD [15–17].

Our findings provide implications for hearing-related urban planning and public health policy. Rural counties with lower PE densities currently exhibit relatively high HD rates, consistent with previous studies showing limited hearing-health infrastructure, reduced healthcare accessibility, and lower availability of hearing-related services in rural communities [29,46,47]. These disparities suggest the importance of improving access to hearing screening, audiological care, rehabilitation resources, and hearing-health education in underserved regions. At the same time, the observed increasing HD-rate trends in counties characterized by denser built environments suggest that urban populations may also face growing hearing-health risks associated with concentrated activity environments and potential long-term environmental noise exposure. These findings highlight the importance of integrating hearing-health considerations into urban planning, occupational safety regulation, transportation planning, and environmental noise management strategies.

This study has limitations. This study remains observational and ecological in nature, and the identified associations are not the direct causal relationships between PE density and HD. The HD data we sourced from the US Census Bureau primarily account for severe hearing impairments or deafness, excluding milder forms of hearing loss. This may underestimate the observed associations between establishment density and overall hearing health. Future work could improve the study by collecting more comprehensive HD data.

## 5 Conclusion

HD is a critical public health issue in the United States, negatively impacting individuals’ quality of life and resulting in substantial economic losses nationwide. This study investigates the association between county-level PE density and HD rate across the U.S. using a combination of spatial and temporal statistical analyses. Our findings are threefold: First, counties with denser PE environments tend to exhibit lower HD rates, and vice versa. Second, after controlling for road networks and sociodemographic factors, PE density is positively associated with HD rate. Third, counties with denser PE environments tend to show a growing trend of HD rate. Taken together, these findings indicate that hearing-health disparities should be understood as both a healthcare access issue and an environmental exposure issue. Rural counties with higher HD rates may require improved access to hearing screening, audiological care, and related healthcare services, while more urbanized counties may require greater attention to occupational and lifestyle-related noise exposure, transportation planning, and environmental noise management. Our findings highlight the importance of integrating hearing-health considerations into urban planning and public health policy.

## Supporting information

S1 Note Identification of hearing-health-risky PEs. (PDF)

S2 Note Robustness analysis on hearing-health-risky PEs. (PDF)

S3 Note Technical details of the soft-DTW implementation. (PDF)
