# Supplementary material for: Spatial and temporal associations between physical establishment density and hearing difficulty across US counties
Source: PLoS One. 2026 Aug 11;21(8):e0355504. doi: 10.1371/journal.pone.0355504 (PMC13460592; doi:10.1371/journal.pone.0355504)
Supplement: Supplementary file 1 [file pone.0355504.s001.pdf]

Spatial and temporal associations between physical establishment density and hearing difficulty across US counties

S1 Note. Identification of hearing-health-risky PEs.

We identified hearing-health-risky establishments based on SafeGraph POI top categories and their corresponding NAICS codes. We grouped the identified establishments into six broader classifications: Entertainment and High-Noise Venues, Manufacturing and Industrial Environments, Mechanical Environments, Construction Environments, Transportation and Logistics, and Energy and Utility Infrastructure. These establishment categories were selected because they may involve elevated noise exposure through occupational activities or lifestyle-related environments[3]. We provided the full list of included categories and NAICS codes in S1 Table.

S1 Table. Category list of hearing-health-risky PEs.

TOP category	NAICS	Classification
Amusement Parks and Arcades	713120	Entertainment and High-Noise Venues
Drinking Places (Alcoholic Beverages)	722410	Entertainment and High-Noise Venues
Performing Arts, Spectator Sports, and Related Industries	711000	Entertainment and High-Noise Venues
Promoters of Performing Arts, Sports, and Similar Events	711300	Entertainment and High-Noise Venues
Other Amusement and Recreation Industries	713900	Entertainment and High-Noise Venues
Aerospace Product and Parts Manufacturing	336400	Manufacturing and Industrial Environments
Architectural and Structural Metals Manufacturing	332300	Manufacturing and Industrial Environments
Beverage Manufacturing	312100	Manufacturing and Industrial Environments
Clay Product and Refractory Manufacturing	327100	Manufacturing and Industrial Environments
Coating, Engraving, Heat Treating, and Allied Activities	332800	Manufacturing and Industrial Environments
Converted Paper Product Manufacturing	322200	Manufacturing and Industrial Environments
Machinery Manufacturing	333000	Manufacturing and Industrial Environments
Fabricated Metal Product Manufacturing	332000	Manufacturing and Industrial Environments
Food Manufacturing	311000	Manufacturing and Industrial Environments
Transportation Equipment Manufacturing	336000	Manufacturing and Industrial Environments

Wood Product Manufacturing	321000	Manufacturing and Industrial Environments
Printing and Related Support Activities	323100	Manufacturing and Industrial Environments
Automotive Repair and Maintenance	811100	Mechanical Environment
Automotive Parts, Accessories, and Tire Stores	441300	Mechanical Environment
Commercial and Industrial Machinery and Equipment Rental and Leasing	532400	Mechanical Environment
Electronic and Precision Equipment Repair and Maintenance	811200	Mechanical Environment
Building Equipment Contractors	238200	Construction Environment
Building Finishing Contractors	238300	Construction Environment
Construction of Buildings	236000	Construction Environment
Heavy and Civil Engineering Construction	237000	Construction Environment
Specialty Trade Contractors	238000	Construction Environment
Couriers and Express Delivery Services	492100	Transportation / Logistics
Truck Transportation	484000	Transportation / Logistics
Warehousing and Storage	493000	Transportation / Logistics
Support Activities for Transportation	488000	Transportation / Logistics
Electric Power Generation, Transmission and Distribution	221100	Energy / Utility Infrastructure
