# Supplementary material for: Spatial and temporal associations between physical establishment density and hearing difficulty across US counties
Source: PLoS One. 2026 Aug 11;21(8):e0355504. doi: 10.1371/journal.pone.0355504 (PMC13460592; doi:10.1371/journal.pone.0355504)
Supplement: Supplementary file 2 [file pone.0355504.s002.pdf]

Spatial and temporal associations between physical establishment density and hearing difficulty across US counties

S2 Note. Robustness analysis on hearing-health-risky PEs.

To evaluate the robustness of the hearing-health-risky PE definition, we conducted a sensitivity analysis using different categories of hearing-health-risky establishments in Models 4–9 (see S2 Table). We separately incorporated Entertainment and High-Noise Venues (abbreviated as EHV in Supplementary Table Y), Manufacturing and Industrial Environments (MIE), Repair and Mechanical Environments (RME), Construction Environments (CE), Transportation and Logistics (TL), and Energy and Utility Infrastructure (EUI) into the regression framework. These establishment categories represent potential noise exposure environments associated with occupational activities or lifestyle-related settings.

S2 Table. Sensitive analysis on hearing-health-risky PE categories.

Variable	Model 4	Model 5	Model 6	Model 7	Model 8	Model 9
EHV density	0.102*<br>(0.040)					
MIE density		0.134**<br>(0.042)				
RME density			0.113<br>(0.071)			
CE density				0.118**<br>(0.041)		
TI density					0.101*<br>(0.038)	
EUI density						0.004<br>(0.012)
Road density	-0.244***<br>(0.067)	-0.284***<br>(0.075)	-0.283*<br>(0.110)	-0.277**<br>(0.086)	-0.244***<br>(0.067)	-0.179**<br>(0.059)
Median Income	-0.211*<br>(0.096)	-0.213*<br>(0.096)	-0.211*<br>(0.096)	-0.215*<br>(0.096)	-0.212*<br>(0.096)	-0.208*<br>(0.096)
Old Dependency Ratio	0.868***<br>(0.095)	0.865***<br>(0.095)	0.874***<br>(0.095)	0.869***<br>(0.095)	0.869***<br>(0.095)	0.880***<br>(0.096)
Sex Ratio	-0.016<br>(0.074)	-0.020<br>(0.074)	-0.016<br>(0.075)	-0.020<br>(0.074)	-0.015<br>(0.074)	-0.011<br>(0.074)
Over Bachelor	-0.531***<br>(0.088)	-0.532***<br>(0.087)	-0.532***<br>(0.088)	-0.533***<br>(0.087)	-0.531***<br>(0.088)	-0.535***<br>(0.088)
Below High School	0.057<br>(0.128)	0.054<br>(0.128)	0.056<br>(0.129)	0.055<br>(0.128)	0.057<br>(0.128)	0.063<br>(0.128)
R2	0.499	0.499	0.500	0.499	0.499	0.498
N	3103	3103	3103	3103	3103	3103

***: p<0.001, **:p<0.01, *:p<0.05

The results show that Entertainment and High-Noise Venues, Manufacturing and Industrial Environments, Construction Environments, and Transportation and Logistics are positively and statistically significantly associated with HD rates after controlling for road density and socioeconomic covariates.
