# Supplementary material for: Spatial and temporal associations between physical establishment density and hearing difficulty across US counties
Source: PLoS One. 2026 Aug 11;21(8):e0355504. doi: 10.1371/journal.pone.0355504 (PMC13460592; doi:10.1371/journal.pone.0355504)
Supplement: Supplementary file 3 [file pone.0355504.s003.pdf]

**Spatial and temporal associations between physical establishment density and hearing difficulty across US counties**

**S3 Note. Technical details of the soft-DTW implementation.**

Based on the silhouette score, the two-cluster solution achieved the highest value (0.318), indicating moderate cluster separation. We selected this solution as a descriptive simplification of temporal patterns. We excluded counties with missing data from the clustering analysis. We standardized the time series before applying soft-DTW K-means clustering and did not apply temporal smoothing. In addition, we tested 5 different random seeds for time-series clustering. The subsequent MW test after each clustering show consistent results, with the increasing trend cluster show statistically significant higher PE density, higher road density, and lower index of relative rurality, demonstrating that our results are stable to different random seeds. In addition, we tested five different random seeds for the time-series clustering analysis. The subsequent MW tests produced consistent results across different clustering runs, with the increasing-trend cluster exhibiting statistically significantly higher PE density, higher road density, and lower relative rurality, indicating that the identified temporal patterns are stable to different random initializations.  (see Supplementary Fig. S3 for clustering results under an alternative random seed). During each test, we implemented soft-DTW K-means clustering with *n_init = 10* and *max_iter = 50* to improve clustering stability across different random initializations.

**S3 Table. Silhouette score for time series clustering on US county hearing difficulty rate.**

| No. of Clusters | Silhouette Scores |
|---|---|
| 2 | 0.318 |
| 3 | 0.233 |
| 4 | 0.189 |
| 5 | 0.187 |
| 6 | 0.113 |
| 7 | 0.111 |

The silhouette ranges from -1 to 1, with a high value indicating more reasonable clustering results. The number of clusters with the highest Silhouette score is optimal for conducting time series clustering.

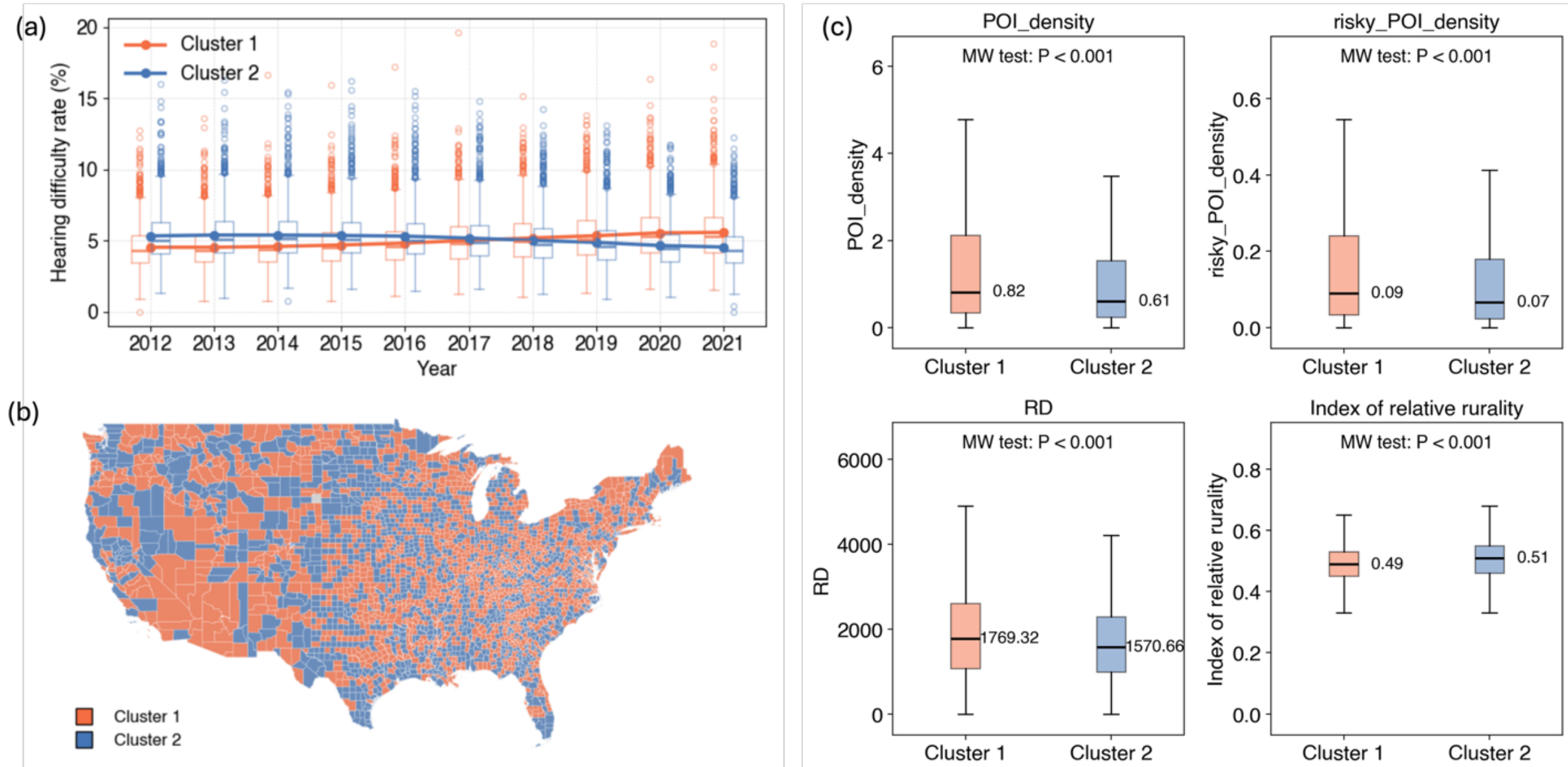


**S1 Figure. Time-series clustering using an alternative random seed.** (a) Temporal clustering results derived from soft Dynamic Time Warping (soft-DTW) K-means clustering. (b) Spatial distribution of the two temporal clusters across U.S. counties. (c) Mann–Whitney U test comparisons between the two clusters.
